# Supplementary material for: The safety and efficacy of remimazolam tosylate combined with propofol in upper gastrointestinal endoscopy: A multicenter, randomized clinical trial
Source: PLoS One. 2023 Aug 3;18(8):e0282930. doi: 10.1371/journal.pone.0282930 (PMC10399878; doi:10.1371/journal.pone.0282930)
Supplement: S1 Protocol — (DOCX) [file pone.0282930.s002.docx]

中华国际医学交流基金会

中青年医学研究专项基金项目

项目名称：甲苯磺酸瑞马唑仑应用于无痛消化内镜检查优势挖掘的大样本、多中心、随机、双盲、平行对照临床研究

申请单位： 四川省人民医院

项目负责人： 杨孟昌

通讯地址： 四川省成都市一环路西二段32号

邮政编码： 610072

联系电话： 18140049936

电子邮箱： ymc681@126.com

| **1.研究内容、研究方法、技术路线、设计方案**  研究目的：探索注射用甲苯磺酸瑞马唑仑用于无痛消化内镜诊疗的优势所在  研究对象：接受无痛胃镜检查的患者  研究设计：采用大样本、多中心、随机、双盲、阳性药物平行对照试验设计  预期样本量：根据我们前期研究，单独使用瑞马唑仑组低血压发生率为18%，单独使用丙泊酚组为40%，瑞马唑仑联合丙泊酚组为25%，采用双侧检验α取0.05，把握度（1-β）为80%，计算样本量为229人，考虑每组脱落率20%，最终计算得出样本量为287。三组采用1：1：1设计，每组均为96例。  **研究方法：**  **随机流程**  受试者在胃镜诊疗开始前2小时，按照 1:1：1 进行随机分组，接受甲苯磺酸瑞马唑仑或者丙泊酚或甲苯磺酸瑞马唑仑联合丙泊酚（以下简称联合组）静脉注射。本试验采用中心化随机分组的方法。参加本试验的各试验中心研究人员在筛选出受试者后，经该中心研究者确认后登录随机系统，填写筛选资料，获取随机号信息，按随机号发放相应的研究药物。  **盲法流程**  随机遮蔽号由 SAS 软件产生，以随机遮蔽号作为总盲底对研究药物编号并导入四川省人民医院中心化随机分组系统。  考虑到本次试验药物甲苯磺酸瑞马唑仑（粉针）和对照药丙泊酚（乳状液体） 外观差距比较大，故本研究设立评价研究者和给药研究者，整个试验过程除了对受试者设盲外，同时也对评价研究者设盲。  本试验设立评价研究者和给药研究者，给药研究者仅参与随机分组、配药及给药过程，其余之外的受试者知情同意过程、筛选、疗效指标和安全性情况的评价、计划外访视等过程均由评价研究者完成。  **入选标准：**   1. 拟进行无痛胃镜的患者； 2. 年龄在 18 - 80 周岁患者，性别不限； 3. 18＜BMI＜30 kg/m^2^ ； 4. 美国麻醉医师协会（ASA）分级为 I～Ⅱ级；   **排除标准：**   1. 术前高血压患者>180/110 mm Hg 或低血压患者<80/50 mm Hg； 2. 心率<50 次/min； 3. 2周内有呼吸道急性炎症且未治愈病史；有严重的心、脑、肺、肝、肾和糖尿病等代谢疾病者；既往有心梗、严重心肌缺血、重度房室传导阻滞；   4.可能发生或曾发生困难气道或有异常手术麻醉恢复史者；  5.有高钾血症等明显电解质紊乱患者；  6.长期使用激素等免疫抑制剂或有肾上腺皮质抑制病史者；  7.已知对乳剂、阿片类药物过敏者；  8.术前内合并使用其它镇静、镇痛类药物者（包括注射、口服及使用相关中成药）；  9. 怀疑有滥用麻醉性镇痛药或镇静药者；  10.有神经肌肉系统疾病、精神疾病者等不配合无法沟通者；  **干预措施**  先将芬太尼 0.5μg/kg用生理盐水稀释至 10 ml，提前 4 min 缓慢静推，1 min 内静推完毕，立即使用甲苯磺酸瑞马唑仑或丙泊酚或联合组进行镇静诱导，受试者达到足够镇静后，即开始胃镜进镜操作。在操作过程中评价研究者根据 MOAA/S 评分情况告知给药研究者按方案给镇静药物（甲苯磺酸瑞马唑仑或丙泊酚）进行镇静维持。另外，在芬太尼给药时给予受试者吸氧（4~6L/min）直到受试者术后完全苏醒。  **甲苯磺酸瑞马唑仑组**：胃镜进镜前镇静诱导：甲苯磺酸瑞马唑仑初始给药剂量为0.2mg/kg，静脉注射时间为约30s。在初始剂量给药结束后 1 分钟内（包括1分钟）：如果受试者达到足够镇静（MOAA/S 评分≤1 分），即开始胃镜进镜操作；如果受试者MOAA/S 评分＞1 分或 MOAA/S 评分≤1 分但尝试胃镜进镜失败，在初始剂量给药结束 1 分钟后允许甲苯磺酸瑞马唑仑追加给药。  胃镜进镜后镇静维持：在胃镜进镜后，为了维持一定的镇静程度（MOAA/S 评分≤1 分），在必要时允许甲苯磺酸瑞马唑仑追加给药。  甲苯磺酸瑞马唑仑追加给药：追加剂量为 2.5mg/次，静脉 bolus 给药。  **丙泊酚组**：胃镜进镜前镇静诱导：丙泊酚初始给药剂量为 2mg/kg静脉注射时间为30s。在初始剂量给药结束后 1 分钟内（包括 1 分钟）：如果受试者达到足够镇静（MOAA/S 评分≤1 分），即开始胃镜进镜操作；如果受试者MOAA/S 评分＞1 分或 MOAA/S 评分≤1 分但尝试胃镜进镜失败，在初始剂量给药结束 1 分钟后允许丙泊酚追加给药。  胃镜进镜后镇静维持：在胃镜进镜后，为了维持一定的镇静程度（MOAA/S 评分≤1 分），在必要时允许丙泊酚追加给药。  丙泊酚追加给药：追加剂量为 0.5mg/kg/次，静脉 bolus 给药。  **联合组：**胃镜进镜前镇静诱导：甲苯磺酸瑞马唑仑初始给药剂量为0.1mg/kg，丙泊酚给药为0.5mg/kg，静脉注射时间为30s。在初始剂量给药结束后 1 分钟内（包括 1 分钟）：如果受试者达到足够镇静（MOAA/S 评分≤1 分），即开始胃镜进镜操作；如果受试者MOAA/S 评分＞1 分或 MOAA/S 评分≤1 分但尝试胃镜进镜失败，在初始剂量给药结束 1 分钟后允许甲苯磺酸瑞马唑仑追加给药。  丙泊酚追加给药：追加剂量为 0.5mg/kg，静脉 bolus 给药。  **呼吸循环的监测与维持**  所有胃肠镜检查均取左侧卧位，患者血压计均绑在右手；  操作中心率<50次/min，酌情静脉注射适量阿托品；  操作中收缩压（SBP）<术前70%或90mmHg，静脉快速补充晶体液200 ml，必要时可静脉注射麻黄碱5-10 mg/次；  操作过程中患者持续吸氧，若脉搏血氧饱和度（SpO2）<95%、时间>30s，手托患者下颌，以改善通气，观察并记录氧饱和度的变化；如<85%，经麻醉机或简易呼吸囊面罩辅助通气，观察并记录氧饱和度的变化过程。  **资料收集**  从开始静推试验药物起计时，记录患者镇静成功时间、内镜置入时间（即为手术开始时间）、追加药物时间、内镜取出时间（即手术结束时间，北京时间，内镜取出时用计时器开始计时 10 min、15 min、20 min）、唤醒时间（以中等音量唤醒指令+轻拍肩膀唤醒）、应答时间（能自报姓名或生日）、自如活动（自行坐起或走动）时间和离院时间、记录术中所用药品剂量及药物追加原因。  血压、心率、呼吸及血氧监测：术前30分钟进行3次测量，取平均值作为基线值。  静推试验药物后测定血压、心率、呼吸及血氧饱和度，**之后设定每2 min测定一次记录直到手术结束**；并且手术结束时（内镜退出时）、苏醒时、手术结束后每10 min测定患者血压并记录直到**受试者满足 Aldrete 评分为≥9 分达到离院标准**。  是否进行辅助呼吸等急救处理，有处理须记录  苏醒质量评分：  **Steward 清醒评分**  **手术结束后 10 min、15 min、20 min、30min进行Steward 清醒评分。**   \|  \| 手术结束后 10 min \| 手术结束后 15 min \| 手术结束后 20 min \| 手术结束后30min \| \| --- \| --- \| --- \| --- \| --- \| \| 清醒  程度 \| - 2=完全清醒 - 1=对刺激有反应 - 0=对刺激物反应 \| - 2=完全清醒 - 1=对刺激有反应 - 0=对刺激物反应 \| - 2=完全清醒 - 1=对刺激有反应 - 0=对刺激物反应 \| - 2=完全清醒 - 1=对刺激有反应 - 0=对刺激物反应 \| \| 呼吸顺畅度 \| - 2=可按医师吩咐咳嗽 - 1=可自主维持呼吸道顺畅 - 0=呼吸道需予以支持 \| - 2=可按医师吩咐咳嗽 - 1=可自主维持呼吸道顺畅 - 0=呼吸道需予以支持 \| - 2=可按医师吩咐咳嗽 - 1=可自主维持呼吸道顺畅 - 0=呼吸道需予以支持 \| - 2=可按医师吩咐咳嗽 - 1=可自主维持呼吸道顺畅 - 0=呼吸道需予以支持 \| \| 肢体活动程度 \| - 2=肢体能做有意识的活动 - 1=可自主维持呼吸道顺畅 - 0=呼吸道需予以支持 \| - 2=肢体能做有意识的活动 - 1=可自主维持呼吸道顺畅 - 0=呼吸道需予以支持 \| - 2=肢体能做有意识的活动 - 1=可自主维持呼吸道顺畅 - 0=呼吸道需予以支持 \| - 2=肢体能做有意识的活动 - 1=可自主维持呼吸道顺畅 - 0=呼吸道需予以支持 \| \| 总分 \|  \|  \|  \|  \|  定向力评分  \| **评分标准** \| 手术结束后  10 min \| \| 手术结束后  15min \| \| 手术结束后  20 min \| \| 手术结束后30min \| \| \| --- \| --- \| --- \| --- \| --- \| --- \| --- \| --- \| --- \| \| 1 分 \| 0 分 \| 1 分 \| 0 分 \| 1分 \| 0 分 \| 1 分 \| 0 分 \| \| **今年是哪一年 ?** \| □ \| □ \| □ \| □ \| □ \| □ \| □ \| □ \| \| **现在是什么季节？** \| □ \| □ \| □ \| □ \| □ \| □ \| □ \| □ \| \| **现在是几月份？** \| □ \| □ \| □ \| □ \| □ \| □ \| □ \| □ \| \| **今天是几号？** \| □ \| □ \| □ \| □ \| □ \| □ \| □ \| □ \| \| **今天是星期几？** \| □ \| □ \| □ \| □ \| □ \| □ \| □ \| □ \| \| **你住在那个省？** \| □ \| □ \| □ \| □ \| □ \| □ \| □ \| □ \| \| **你住在那个县（区）？** \| □ \| □ \| □ \| □ \| □ \| □ \| □ \| □ \| \| **你住在那个乡（街道）？** \| □ \| □ \| □ \| □ \| □ \| □ \| □ \| □ \| \| **咱们现在在那个医院？** \| □ \| □ \| □ \| □ \| □ \| □ \| □ \| □ \| \| **咱们现在在第几层楼？** \| □ \| □ \| □ \| □ \| □ \| □ \| □ \| □ \| \| **总计** \|  \| \|  \| \|  \| \|  \|  \|  抬头评估（头颈部肌力）  \| **抬头评估** \| \| \| \| \| --- \| --- \| --- \| --- \| \| 是否完成了抬头评估：□_1_ 是 □_2_ 否 \| \| \| \| \| 若否，未查原因：_____________________ \| \| \| \| \| 若是，评估日期：\|___\|____\|____\|___\|年\|___\|___\|月\|___\|___\|日 \| \| \| \| \| 若是，请完成如下评分（单选） \| \| \| \| \| 时间点 \| 检测时间 \| 检测结果 \| 肌力分级评定标准  0级 可自主抬头保持动作10s以上  1级 可自主抬头保持动作5-10s  2级 可自主抬头保持动作小于5秒，记录保持时间  3级 无法自主抬头 \| \| 麻醉前基线测量 \| \|___\|___\|:\|___\|___\| \| □_0_ □_1_ □_2，_可保持 秒 □_3_ \| \| \| 术后10min \| \|___\|___\|:\|___\|___\| \| □_0_ □_1_ □_2，_可保持 秒 □_3_ \| \| \| 术后20min \| \|___\|___\|:\|___\|___\| \| □_0_ □_1_ □_2，_可保持 秒 □_3_ \| \| \| 术后30min \| \|___\|___\|:\|___\|___\| \| □_0_ □_1_ □_2，_可保持 秒 □_3_ \| \|  Bromage改良法测下肢肌力  \| Bromage改良法（主要观察指标） \| \| \| \| \| --- \| --- \| --- \| --- \| \| 是否完成了下肢肌力的Bromage改良法分级：□_1_ 是 □_2_ 否 \| \| \| \| \| 若否，未查原因：_____________________ \| \| \| \| \| 若是，评估日期：\|___\|____\|____\|___\|年\|___\|___\|月\|___\|___\|日 \| \| \| \| \| 若是，请完成如下评分（单选） \| \| \| \| \| 时间点 \| 检测时间 \| 检测结果 \| Bromage肌力分级评定标准  0级 无运动阻滞  1级 膝关节、踝关节能运动(不能抬起大腿)  2级 仅能活动踝关节  3级 踝、膝、髋关节均不能转动 \| \| 麻醉前基线测量 \| \|___\|___\|:\|___\|___\| \| □_0_ □_1_ □_2_ □_3_ \| \| \| 术后10min \| \|___\|___\|:\|___\|___\| \| □_0_ □_1_ □_2_ □_3_ \| \| \| 术后20min \| \|___\|___\|:\|___\|___\| \| □_0_ □_1_ □_2_ □_3_ \| \| \| 术后30min \| \|___\|___\|:\|___\|___\| \| □_0_ □_1_ □_2_ □_3_ \| \|  共济失调表现量表（术后20-30min） 1. 坐姿 (双臂交叉,双大腿并拢,坐在硬座上)  0 = 正常  1 = 躯干轻度摇晃  2 = 躯干和腿中度摇晃  3 = 严重的不平衡  4 = 不能坐  评分 右 左  2. 指指试验(动作震颤和/ 或不稳定性) (患者坐位,在胸前高度,相距1cm ,作匀速对指(示指) 动作10 秒。要求患者睁眼控制动作)  0 = 正常  1 = 轻度不稳  2 = 中等程度的摇摆,幅度< 10cm  3 = 手指相当大的摇摆,幅度在10cm 和40cm之间  4 = 冲撞样运动,幅度> 40cm  评分 右 左  3. 指鼻试验: 手指意向性震颤 (在投掷样运动阶段出现。患者坐在适合的椅子上,每次测试前手放在大腿上。要求患者目光控制动作。每侧肢体检查3 次)  0 = 正常  1 = 动作轻度偏差  2 = 中等程度震颤,幅度< 10cm  3 = 震颤,幅度在10cm 和40cm 之间  4 = 严重的震颤,幅度> 40cm  评分 右 左  4. 睁眼,双脚并立身体摇晃程度  0 = 正常  1 = 轻度晃动  2 = 明显晃动(在头部水平< 10cm)  3 = 严重的晃动(在头部水平> 10cm) ,有摔倒危险  4 = 立即摔倒  评分 右 左  5. 闭眼,双脚并立身体摇晃程度  0 = 正常  1 = 轻度晃动  2 = 明显晃动(在头部水平< 10cm)  3 = 严重的晃动(在头部水平> 10cm) ,有摔倒危险  4 = 立即摔倒  评分 右 左  6. 行走能力 (观察靠墙约1.5 米的行走能力,包括转身动作)  0 = 正常  1 = 接近正常,但不能两脚一前一后在一条直线上行走  2 = 行走不需要扶助,但明显异常  3 = 行走不需要扶助,但摇晃明显,转身困难  4 = 不能独立行走,在行走10 米的测试中间断,需要扶墙  5 = 需借助一个拐杖行走  6 = 需借助两个拐杖或助行器行走  7 = 需陪人扶助行走  8 = 即使在陪人帮助下也不能行走(日常活动限于轮椅)  总分  安全性评价：  观察所有受试者在临床研究期间发生的任何不良事件，包括临床症状及生命体征异常、实验室检查中出现的异常，记录其临床表现特征、严重程度、发生时间、持续时间、处理方法及预后，并判定其与试验药物之间的相关性。  不良事件包括但不限于下表： 不良事件记录及处理 无 □ 有 □（请填写不良事件报告表）   \|  \| 发生时间 \| 不良事件表现 \| 处理方法 \| 处理效果  （是否改善） \| 与研究药物的关系 \| \| --- \| --- \| --- \| --- \| --- \| --- \| \| 上呼吸道梗阻 \| \|_\|_\|:\|_\|_\|^’^\|_\|_\|^”^ \| - 0 级：无上呼吸道梗阻； - 1 级：轻微鼾声但吸气正常； - 2 级：很强的鼾声或有吸气凹陷，但能保证通气正常； - 3 级：必须依赖口咽通气道   或托下颌才能缓解。 \| 否□是□ 处理： \| 是 □  否 □ \| □肯定有关  □很可能有关  □可能有关  □可能无关  □肯定无关 \| \| 低氧血症 \| \|_\|_\|:\|_\|_\|^’^\|_\|_\|^”^ \| □1 级：96%≤SpO_2_≤100%；  □2 级：91%≤SpO_2_≤95%；  □3 级：86%≤SpO_2_≤90%；  □4 级：SpO_2_≤85% \| 否□是□ 处理： \| 是 □  否 □ \| □肯定有关  □很可能有关  □可能有关  □可能无关  □肯定无关 \| \| 呼吸暂停 \| \|_\|:\|_\|_\|^’^\|_\|_\|^”^ \| □0：呼吸频率≥8 次/ 分；  □1：呼吸频率4-8次/分，> 20 s，呼吸频率＜3 次/分；  **发生次数 \|_\|次** \| 否□ 是□ 处理： \| 是 □  否 □ \| □肯定有关  □很可能有关  □可能有关  □可能无关  □肯定无关 \| \| 心率与心律变化 \| \|_\|_\|:\|_\|_\|^’^\|_\|_\|^”^ \| □ 0：无，心率50-100次/分  □1：心率45-50次/分,100-120 次/分或出现心律失常；  □ 2 ：心 率 < 4 5 次 / 分 ，>120次/分  **发生次数 \|_\|次** \| 否□ 是□ 处理： \| 是 □  否 □ \| □肯定有关  □很可能有关  □可能有关  □可能无关  □肯定无关 \| \| 低血压 \| \|_\|_\|:\|_\|_\|^’^\|_\|_\|^”^ \| □ 0：无；  □ 1：SBP<术前 70％或 90 mmHg；  **发生次数 \|_\|次** \| 否□是□ 处理： \| 是 □  否 □ \| □肯定有关  □很可能有关  □可能有关  □可能无关  □肯定无关 \|  \| 肌颤 \| \|_\|_\|:\|_\|_\|^’^\|_\|_\|^”^ \| - 1 分：无肉眼可见的肌束收缩； - 2 分：肢体末端微弱收缩； - 3 分：面部躯干四肢肌肉轻度收缩； - 4 分：面部躯干四肢肌肉   强烈收缩，甚至伴有肢体扭动； \| 否□是□ 处理： \| 是 □  否 □ \| □肯定有关  □很可能有关  □可能有关  □可能无关  □肯定无关 \| \| --- \| --- \| --- \| --- \| --- \| --- \| \| 恶心呕吐 \| \|_\|_\|:\|_\|_\|^’^\|_\|_\|^”^ \| - 1 分：无恶心呕吐； - 2 分：轻度恶心，腹部不适，但无呕吐； - 3 分：恶心呕吐明显， 但无内容物吐出； - 4 分：严重的呕吐，有胃   液等内容物吐出且需要药物控制； \| 否□ 是□ 处理： \| 是 □  否 □ \| □肯定有关  □很可能有关  □可能有关  □可能无关  □肯定无关 \| \| 体 动 （非肌 颤 因素 引 起的 肢 体活 动） \| \|_\|_\|:\|_\|_\|^’^\|_\|_\|^”^ \| □ 0 分：无；   - 1 分：一般体动，脚趾动、手动，不影响检查的体动； - 2 分：严重体动，腿动或臀动，影响检查的体动； \| 否□是□ 处理： \| 是 □  否 □ \| □肯定有关  □很可能有关  □可能有关  □可能无关  □肯定无关 \| \| 其 他：如红 斑、发热 、 流 汗、面色苍白等， 请具体描述 \| \|_\|_\|:\|_\|_\|^’^\|_\|_\|^”^ \| □ 0：无  □ 1：有  描述： \| 否□是□ 处理： \| 是□ 否 □ \| □肯定有关  □很可能有关  □可能有关  □可能无关  □肯定无关 \|   **合并用药与禁用药**  受试者使用试验药物和试验辅助用药（利多卡因胶浆、芬太尼）以外的任何药物均属于合并用药，受试者在随机开始前 14天内、以及在整个研究过程中使用的所有药物治疗必须记录。  禁用药   \| 药物种类 \| 药物名称 \| 最短洗脱  期 \| \| --- \| --- \| --- \| \| 镇静催眠类药物 \| 苯二氮卓类(三唑仑、安定、咪达唑仑等)、 非苯二氮卓类（唑吡坦、佐匹克隆、扎来普隆等） \| ≥7 天 \| \| 麻醉镇静类药物 \| 丙泊酚、七氟烷、麻醉乙醚、氧化亚氮、硫喷  妥钠、氯胺酮、依托咪酯、羟丁酸钠等 \| ≥7 天 \| \| 镇痛类药物 \| 吗啡、舒芬太尼、瑞芬太尼、芬太尼、美沙酮、  可待因、喷他佐辛、曲马多等 \| ≥7 天 \| \| 局部麻醉药 \| 利多卡因、普鲁卡因、苯佐卡因、布比卡因、  达克罗宁等 \| ≥7 天 \|   研究持续时间：每个参与研究的患者将于术前采集基线资料并被随访至手术后48小时。  研究结束  研究结束定义为“最后一位患者最后一次随访结束”。 |
| --- | --- | --- | --- | --- | --- | --- | --- | --- | --- | --- | --- | --- | --- | --- | --- | --- | --- | --- | --- | --- | --- | --- | --- | --- | --- | --- | --- | --- | --- | --- | --- | --- | --- | --- | --- | --- | --- | --- | --- | --- | --- | --- | --- | --- | --- | --- | --- | --- | --- | --- | --- | --- | --- | --- | --- | --- | --- | --- | --- | --- | --- | --- | --- | --- | --- | --- | --- | --- | --- | --- | --- | --- | --- | --- | --- | --- | --- | --- | --- | --- | --- | --- | --- | --- | --- | --- | --- | --- | --- | --- | --- | --- | --- | --- | --- | --- | --- | --- | --- | --- | --- | --- | --- | --- | --- | --- | --- | --- | --- | --- | --- | --- | --- | --- | --- | --- | --- | --- | --- | --- | --- | --- | --- | --- | --- | --- | --- | --- | --- | --- | --- | --- | --- | --- | --- | --- | --- | --- | --- | --- | --- | --- | --- | --- | --- | --- | --- | --- | --- | --- | --- | --- | --- | --- | --- | --- | --- | --- | --- | --- | --- | --- | --- | --- | --- | --- | --- | --- | --- | --- | --- | --- | --- | --- | --- | --- | --- | --- | --- | --- | --- | --- | --- | --- | --- | --- | --- | --- | --- | --- | --- | --- | --- | --- | --- | --- | --- | --- | --- | --- | --- | --- | --- | --- | --- | --- | --- | --- | --- | --- | --- | --- | --- | --- | --- | --- | --- | --- | --- | --- | --- | --- | --- | --- | --- | --- | --- | --- | --- | --- | --- | --- | --- | --- | --- | --- | --- | --- | --- | --- | --- | --- | --- | --- | --- | --- | --- | --- | --- | --- | --- | --- | --- | --- | --- | --- | --- | --- | --- | --- | --- | --- | --- | --- | --- | --- | --- | --- | --- | --- | --- | --- | --- | --- | --- | --- | --- | --- | --- | --- | --- | --- | --- | --- | --- | --- | --- | --- | --- | --- | --- | --- | --- | --- | --- | --- |
