# Supplementary material for: The safety and efficacy of remimazolam tosylate combined with propofol in upper gastrointestinal endoscopy: A multicenter, randomized clinical trial
Source: PLoS One. 2023 Aug 3;18(8):e0282930. doi: 10.1371/journal.pone.0282930 (PMC10399878; doi:10.1371/journal.pone.0282930)
Supplement: S2 Protocol — (PDF) [file pone.0282930.s003.pdf]

China International Medical Exchange Foundation

Special Fund for Young and Middle-aged Medical Research Project

Project Name: The application of remimazolam tosylate in painless gastrointestinal endoscopy: A large sample, multicenter, randomized, double-blind, parallel-controlled clinical study

Application Unit: Sichuan Provincial People's Hospital

Project Manager: Mengchang Yang

Address: No. 32, West Second Section of First Ring Road, Chengdu,

Postal Code: 610072

Tel: 18140049936

Email: ymc681@126.com

1. Research content, method, technical route, design plan

Aim of the study: To explore the advantages of using remimazolam tosylate for injection in painless gastrointestinal endoscopy

Study population: Patients undergoing painless gastroscopy

Study design: Large sample, multicenter, randomized, double-blind, positive drug parallel controlled trial design was used

Sample size: According to our preliminary study, the incidence of hypotension was 18% in the remimazolam alone group, 40% in the propofol alone group, and 25% in the remimazolam combined with propofol group, using a two-sided test with α taken as 0.05 and a certainty (1-β) of 80%, the calculated sample size was 229, considering a 20% shedding rate in each group, the final calculated sample size was 287. three groups using a 1 : 1:1 design with 96 cases in each group.

Methods

Randomization

Subjects were randomized 2 hours prior to the start of the gastroscopy consultation according to a 1:1:1 randomization group to receive either remimazolam tosylate or propofol or remimazolam tosylate combined with propofol (hereafter referred to as the R+P group) intravenously. This trial was conducted using a centralized randomized grouping approach. After the subjects were screened by the investigators of each trial center participating in this trial, they logged into the randomization system after confirmation by the investigator of that center, filled in the screening information, obtained the randomization number information, and were issued the corresponding study drug according to the randomization number.

Blinding

The random masking numbers were generated by SAS software, and the random masking numbers were used as the total blind base to number the study drugs and imported into the centralized random grouping system of Sichuan Provincial People's Hospital.

Considering the large difference in appearance between the trial drug rimazolam mesylate (powder) and the control drug propofol (emulsion), an evaluation investigator and an administration investigator were established for this study, and the entire trial was blinded to the subjects as well as to the evaluation investigator.

The evaluation investigator and the dosing investigator were established for this trial. The dosing investigator was only involved in the randomization grouping, dosing and administration process, while the rest of the process, such as informed consent process, screening, evaluation of efficacy indicators and safety profile, and unplanned visits, were completed by the evaluation investigator.

Inclusion criteria.

1. Patients who are to undergo painless gastroscopy.
2. Patients between the ages of 18-80 years, regardless of gender.
3. 18 < BMI < 30 kg/m^2.
4. American Society of Anesthesiologists (ASA) classification of I to II.

Exclusion criteria.

1. Preoperative hypertensive patients >180/110 mmHg or hypotensive patients <80/50 mm Hg.
2. Heart rate <50 beats/min.
3. A history of acute inflammation of the respiratory tract that has not been cured within 2 weeks; Severe metabolic diseases of the heart, brain, lungs, liver, kidneys and diabetes; previous heart attack, severe myocardial ischemia, severe atrioventricular block.
4. Those who may have or have had a difficult airway or a history of recovery from abnormal surgical anesthesia.
5. Patients with obvious electrolyte disturbances.
6. Long-term use of immunosuppressive agents such as hormones or a history of adrenal cortical suppression.
7. Persons with known allergy to emulsions, opioids
8. Preoperative combined use of other sedative and analgesic drugs (including injections, oral and use of related proprietary Chinese medicines).
9. Persons suspected of abusing narcotic analgesics or sedative drugs.
10. People with neuromuscular diseases, mental illnesses, etc. who do not cooperate and cannot communicate.

Intervention

Fentanyl 0.5 μg/kg was first diluted to 10 ml with saline and slowly sedated 4 min in advance, and the sedation was completed within 1 min. Sedation was immediately induced with either remimazolam mesylate or propofol or a combination group, and the gastroscopic approach was started as soon as the subject achieved adequate sedation. During the operation

the evaluation investigator informed the dosing investigator to administer the sedative drug (remimazolam tosylate or propofol) for sedation maintenance according to the protocol based on the MOAA/S score. In addition, the subject was given oxygen (4-6 L/min) while fentanyl was administered until the subject was fully awake after the procedure.

Remimazolam tosylate group: sedation induction prior to gastroscopy: remimazolam tosylate was administered at an initial dose of 0.2 mg/kg for approximately 30 s. Within 1 minute of the initial dose (including 1 minute): if the subject achieved adequate sedation (MOAA/S score ≤1), gastroscopy was initiated; if the subject achieved a MOAA/S score >1 or a MOAA/S score ≤1 but failed to attempt gastroscopy, additional doses of remimazolam tosylate were allowed 1 minute after the initial dose. If the subject has a MOAA/S score >1 or a MOAA/S score ≤1 but failed to attempt gastroscopic access, additional dosing of remimazolam tosylate was allowed 1 minute after the initial dose was administered.

Maintenance of sedation after gastroscopy access: To maintain a certain level of sedation (MOAA/S score ≤1) after gastroscopy access, additional dosing of remimazolam tosylate is allowed when necessary.

Remimazolam tosylate additional dose: additional dose of 2.5 mg administered intravenously bolus.

Propofol group: sedation induction prior to gastroscopy: propofol initial dose of 2 mg/kg given intravenously for 30 s. Within 1 minute of the end of the initial dose (including 1 minute): if the subject achieves adequate sedation (MOAA/S score ≤1), gastroscopy access is initiated; if the subject If the subject achieves a MOAA/S score >1 or a MOAA/S score ≤1 but fails to attempt gastroscopic access, additional propofol is allowed 1 minute after the initial dose is administered.

Post-gastroscopy sedation maintenance: To maintain a certain level of sedation after gastroscopy access (MOAA/S score ≤1), additional propofol administration was allowed when necessary.

Additional propofol administration: additional dose of 0.5mg/kg, administered intravenously bolus.

R+P group: sedation induction prior to gastroscopy: initial dose of 0.1 mg/kg for remimazolam tosylate and 0.5 mg/kg for propofol administered intravenously for 30s. Within 1 minute of the initial dose (including 1 minute): if the subject achieves adequate sedation (MOAA/S score ≤1), gastroscopy access is initiated; if the subject achieves a MOAA/S score >1 or a MOAA/S score ≤1 but fails the gastroscopy access attempt, additional doses of

remimazolam tosylate are allowed 1 minute after the initial dose is administered. If the subject has a MOAA/S score >1 or a MOAA/S score ≤1 and failed gastroscopy access attempt, additional dosing of remimazolam tosylate was allowed 1 minute after the initial dose.

Additional propofol administration: additional dose of 0.5mg/kg, administered intravenously bolus.

Monitoring and maintenance of respiratory circulation

All gastroscopies were performed in the left lateral position, with the patient's sphygmomanometer strapped to the right hand.

Operate at a central rate of <50 beats/min and administer an appropriate amount of intravenous atropine, as appropriate.

Intraoperative systolic blood pressure (SBP) < 70% or 90mmHg preoperatively, rapid intravenous supplemental crystalloid 200 ml and, if necessary, intravenous ephedrine 5-10 mg/time.

During the operation, the patient was continuously oxygenated. If the pulse oximetry (SpO_2) was <95% for >30s, the patient's jaw was held by hand to improve ventilation, and the change in oxygen saturation was observed and recorded; if <85%, ventilation was assisted by an anesthesia machine or a simple respiratory bag mask, and the course of oxygen saturation was observed and recorded.

Data Collection

The time of successful sedation, time of endoscope insertion (i.e., time of procedure start), time of additional medication, time of endoscope removal (i.e., time of procedure end, Beijing time, 10 min, 15 min, 20 min by timer at endoscope removal), time of wake-up (wake-up command at medium volume + tap on shoulder), time of response (able to (able to give his or her name or birthday), time to move freely (sitting up or walking on his or her own) and time to leave the hospital, and record the dose of drugs used during the operation and the reason for additional drugs.

Blood pressure, heart rate, respiration and oxygen monitoring: 3 measurements were taken 30 minutes before the procedure and the average value was taken as the baseline value.

Blood pressure, heart rate, respiration, and oxygen saturation were measured after intravenous drug administration and recorded every 2 min until the end of the procedure; and blood pressure was measured and recorded at the end of the procedure (when the endoscope was withdrawn), during

awakening, and every 10 min after the procedure until the subject met the criteria for discharge with an Aldrete score of ≥9.

Whether to carry out first aid treatment such as assisted breathing, there must be records of treatment

Wake quality rating.

Steward Score

The Steward Score was performed at 10 min, 20min,30min after the end of the procedure.

	10 min after the end of the procedure	15 min after the end of the procedure	20 min after the end of the procedure	30min after the end of surgery
Sobriety Degree	☐ 2 = Fully awake<br>☐ 1=responsive to stimuli<br>☐ 0=response to stimulus	☐ 2 = Fully awake<br>☐ 1=responsive to stimuli<br>☐ 0=response to stimulus	☐ 2 = Fully awake<br>☐ 1=responsive to stimuli<br>☐ 0=response to stimulus	☐ 2 = Fully awake<br>☐ 1=responsive to stimuli<br>☐ 0=response to stimulus
Smoothness of breathing	☐ 2 = May cough as ordered by physician<br>☐ 1=Able to maintain a smooth airway on its own<br>☐ 0 = respiratory tract needs to be supported	☐ 2 = May cough as ordered by physician<br>☐ 1=Able to maintain a smooth airway on its own<br>☐ 0 = respiratory tract needs to be supported	☐ 2 = May cough as ordered by physician<br>☐ 1=Able to maintain a smooth airway on its own<br>☐ 0 = respiratory tract needs to be supported	☐ 2 = May cough as ordered by physician<br>☐ 1=Able to maintain a smooth airway on its own<br>☐ 0 = respiratory tract needs to be supported
Degree of physical activity	☐ 2=Limbs can do conscious activities<br>☐ 1=Able to maintain a smooth airway on its own<br>☐ 0 = respiratory tract needs to be supported	☐ 2=Limbs can do conscious activities<br>☐ 1=Able to maintain a smooth airway on its own<br>☐ 0 = respiratory tract needs to be supported	☐ 2=Limbs can do conscious activities<br>☐ 1=Able to maintain a smooth airway on its own<br>☐ 0 = respiratory tract needs to be supported	☐ 2=Limbs can do conscious activities<br>☐ 1=Able to maintain a smooth airway on its own<br>☐ 0 = respiratory tract needs to be

				supported
Total Score	________________	________________	________________	________________

Orientational force score

Scoring Criteria	After the surgery 10 min		After the surgery 15min		After the surgery 20 min		30min after the end of surgery	
	1 point	0 points	1 point	0 points	1 point	0 points	1 point	0 points
**What year is it ?**	□	□	□	□	□	□	□	□
**What season is it?**	□	□	□	□	□	□	□	□
**What month is it?**	□	□	□	□	□	□	□	□
**What is today's date?**	□	□	□	□	□	□	□	□
**What day of the week is it?**	□	□	□	□	□	□	□	□
**What province do you live in?**	□	□	□	□	□	□	□	□
**Which county (region) do you live in?**	□	□	□	□	□	□	□	□
**Which township (street) do you live in?**	□	□	□	□	□	□	□	□
**Which hospital are we in?**	□	□	□	□	□	□	□	□
**What floor are we on?**	□	□	□	□	□	□	□	□
**Total**								

Head-up assessment (head and neck muscle strength)

**Heads Up Assessment**
Whether a heads-up assessment was completed: □1 Yes □2 No
If no, reason not checked: ______________________
If yes, assessment date: |__| __ __|| __| year|__| __| month|__| __| day
If yes, please complete the following scoring (single choice)

Time Point	Testing time	Test results	Muscle strength grading assessment criteria<br>Level 0 Can hold the action of raising the he independently for more than 10s<br>Level 1 Autonomous head lift to maintain movement f 5-10s<br>Level 2 Autonomous head liftable holding action le than 5 seconds, record holding time<br>Level 3 Unable to lift head voluntarily
Pre-anesthesia baseline measurements	|__| __|:|__| __|	☐0 ☐1 ☐2, may hold seconds ☐3	
10min after surgery	|__| __|:|__| __|	☐0 ☐1 ☐2, may hold seconds ☐3	
20min after surgery	|__| __|:|__| __|	☐0 ☐1 ☐2, may hold seconds ☐3	
30min after surgery	|__| __|:|__| __|	☐0 ☐1 ☐2, may hold seconds ☐3	

Bromage modified method for measuring lower limb muscle strength

Bromage modification method (main observation)

Whether Bromage modified method grading of lower extremity muscle strength was completed: ☐1 Yes No

If no, reason not checked: ______________________

If yes, assessment date: |__| __ __|| __| year|__| __| month|__| __| day

If yes, please complete the following scoring (single choice)

Time Point	Testing time	Test results	Bromage muscle strength grading criteria<br>Grade 0 No motor block<br>Grade 1 Knee and ankle joints can move (cannot l thighs)<br>Grade 2 Only able to move the ankle joint<br>Grade 3 Inability to rotate the ankle, knee and hip joint
Pre-anesthesia baseline measurements	|__| __|:|__| __|	☐0 ☐1 ☐2 ☐3	
10min after surgery	|__| __|:|__| __|	☐0 ☐1 ☐2 ☐3	

20min after surgery	|__| __|:|__| __|	☐0 ☐1 ☐2 ☐3
30min after surgery	|__| __|:|__| __|	☐0 ☐1 ☐2 ☐3

Ataxia Performance Scale (20-30min post-op)

1. Sitting position (arms crossed, thighs together, sitting on the hard seat)

0 = Normal

1 = Mild torso swaying

2 = Moderate swaying of the trunk and legs

3 = Severe imbalance

4 = Cannot sit

Rating Right Left

2. Finger test (tremor and/or instability) (Patients were seated at chest height, 1 cm apart, and performed uniform finger-to-finger movements for 10 seconds. Ask the patient to open his eyes to control the movement)

0 = Normal

1 = Mild instability

2 = Moderate sway, amplitude < 10cm

3 = considerable finger wagging, between 10cm and 40cm in amplitude

4 = Punch-like movement, amplitude > 40cm

Rating Right Left

3. Finger-nose test: Intentional tremor of the fingers (occurs during the throwing-like movement phase. The patient is seated in a suitable chair and the hand is placed on the thigh before each test. The patient is asked to control the movement by gaze. Each limb was examined 3 times)

0 = Normal

1 = Mild deviation in movement

2 = Moderate tremor, amplitude < 10 cm

3 = Tremor, amplitude between 10cm and 40cm

4 = Severe tremor, amplitude > 40 cm

Rating Right Left

4. open eyes, feet together body shaking degree

0 = Normal

1 = Mild shaking

2 = Significant swaying (< 10cm at head level)

3 = Severe swaying (> 10cm at head level), risk of falling

4 = Immediate fall

Rating Right Left

5. eyes closed, feet together body swaying degree

0 = Normal

1 = Mild shaking

2 = Significant swaying (< 10cm at head level)

3 = Severe swaying (> 10cm at head level), risk of falling

4 = Immediate fall

Rating Right Left

6. walking ability (observation of walking ability against the wall of about 1.5 meters, including turning movements)

0 = Normal

1 = close to normal, but cannot walk in a straight line with one foot in front of the other

2 = Walking without assistance, but clearly abnormal

3 = Walking without assistance, but swaying is obvious and turning around is difficult

4 = Cannot walk independently, interrupted in the test of walking 10 meters, need to hold the wall

5 = Need to walk with the help of a cane

6 = Need to walk with two crutches or walkers

7 = Needs a companion to help walk

8 = Unable to walk even with the help of a companion (daily activities are limited to a wheelchair)

Total Score _________

Safety evaluation.

All subjects were observed for any adverse events that occurred during the clinical study, including abnormal clinical symptoms and vital signs, and abnormalities in laboratory tests, and their clinical presentation characteristics, severity, time of occurrence, duration, management and prognosis were recorded, and correlation with the test drug was determined. Adverse events include, but are not limited to, the following table.

Adverse event recording and handling

None □ Yes □ (Please fill in the adverse event report form)

	time	Adverse event performance	Processing method	Treatment effect (whether to improve)	Relationship to study drugs

Upper airway obstruction	|_|_| :|_| _|` |_|_| ,,	☐ Grade 0: no upper airway obstruction.<br>☐ Level 1: slight snoring but normal inspiration.<br>☐ Level 2: very strong snoring or inspiratory depression, but normal ventilation is ensured.<br>☐ Level 3: Must rely on oropharyngeal airway<br>Or support the jaw to get relief.	No□Yes□<br>Disposal.____	Yes □<br>No □	□ Definitely related<br>□ Most likely related<br>□ may be related to<br>□ May not be relevant<br>□ Definitely not related
Hypoxemia	|_|_| :|_| _|` |_|_| ,,	□1 level: 96% ≤ SpO_2 ≤ 100%.<br>□2 level: 91% ≤ SpO_2 ≤ 95%.<br>□ Grade 3: 86% ≤ SpO_2 ≤ 90%.<br>□4 level: SpO_2 ≤85%	No□Yes□<br>Disposal.____	Yes □<br>No □	□ Definitely related<br>□ Most likely related<br>□ may be related to<br>□ May not be relevant<br>□ Definitely not related
Apnea	|_|:| _|_| , |_|_ |"	□0: Respiratory rate ≥ 8 breaths/min.<br>□1: respiratory rate 4-8 breaths/min, > 20 s, respiratory rate <3 breaths/min.	No□<br>Yes□<br>Disposal.____	Yes □<br>No □	□ Definitely related<br>□ Likely related<br>□ may be related to<br>□ May not be relevant<br>□ Definitely not related
Heart rate and rhythm changes	|_|_ |:|_|	□ 0: None, heart rate 50-100 beats/min<br>□1: Heart rate 45-50 beats/min, 100-120 beats/min or	No□<br>Yes□	Yes □<br>No □	□ Definitely related<br>□ Most likely related

	_|'<br>|_|_<br>|"	arrhythmia.<br>□2：Heart rate <45beats/min, or> 120 beats/min	Disposal.____		□ may be related to<br>□ May not be relevant<br>□ Definitely not related
Low blood pressure	|_|_<br>|:|_| _|'<br>|_|_<br>|"	□ 0: none.<br>□ 1: SBP < 70% or 90 mmHg preoperatively.	No□Yes□<br>Disposal.____	Yes □<br>No □	□ Definitely related<br>□ Most likely related<br>□ may be related to<br>□ May not be relevant<br>□ Definitely not related

Myofibrillation	|_|_|:|_<br>|_|'<br>|_|_|"	☐ 1: absence of muscular bundle contraction visible to the naked eye.<br>☐ 2: weak contraction at the end of the limb.<br>☐ 3: Mild contraction of the muscles of the trunk and extremities of the face.<br>☐ 4: facial trunk extremity muscles<br>Intense contractions, even with limb twisting.	No□Yes□<br>Disposal.___	Yes □<br>No □	□ Definitely related<br>□ Likely related<br>□ may be related to<br>□ May not be relevant<br>□ Definitely not related

Nausea and vomiting	|_|_|:|_ |_|' |_|_|"	☐ 1: absence of nausea and vomiting.<br>☐ 2: Mild nausea with abdominal discomfort but no vomiting.<br>☐ 3: nausea and vomiting are evident, but no contents are vomited.<br>☐ 4: severe vomiting with stomach<br>The spitting out of contents such as fluid and the need for medication to control it.	No□<br>Yes□<br>Disposal.__	Yes □<br>No □	□ Definitely related<br>□ Most likely related<br>□ may be related to<br>□ May not be relevant<br>□ Definitely not related
body movements,	|_|_|:|_ |_|' |_|_|"	□ 0 : none.<br>☐ 1: general body movements, toe movements, manual movements, body movements that do not interfere with the examination.<br>☐ 2: severe body movements, leg or hip movements, body movements that interfere with the examination.	No□Yes□<br>Disposal.__	Yes □<br>No □	□ Definitely related<br>□ Most likely related<br>□ may be related to<br>□ May not be relevant<br>□ Definitely not related
Others: Erythema, fever sweating, pale face, etc. Please describe specifically	|_|_|:|_ |_|' |_|_|"	□ 0: None<br>□ 1: Yes<br>Description.________________	No□Yes□<br>Disposal.__	Yes□ No □	□ Definitely related<br>□ Most likely related<br>□ may be related to<br>□ May not be relevant<br>□ Definitely not related

Combination of drugs and prohibited drugs

Subjects using any medication other than the trial drug and trial adjuvant (lidocaine syrup, fentanyl) is considered a combination, and all medication used by the subject within 14 days prior to randomization and throughout the study must be documented.

Prohibited drugs

Drug type	Drug name	Minimum elution Period
Sedative-hypnotic drugs	Benzodiazepines (triazolam, valium, midazolam, etc.), non-benzodiazepines (zolpidem, zopiclone, zaleplon, etc.)	≥7 days
Narcotic sedative drugs	Propofol, sevoflurane, anesthetic ether, nitrous oxide, sulfur spray<br>Sodium Tolstoy, ketamine, etomidate, sodium oxybutyrate, etc.	≥7 days
Analgesic drugs	morphine, sufentanil, remifentanil, fentanyl, methadone<br>Codeine, pentazocine, tramadol, etc.	≥7 days
Local anesthetics	Lidocaine, procaine, benzocaine, bupivacaine<br>Dacronin, etc.	≥7 days

Study duration: Each participating patient will have baseline information collected preoperatively and will be followed up until 48 hours after surgery.

End of study

The end of the study was defined as "the end of the last follow-up visit for the last patient".
