# Supplementary material for: The safety and efficacy of remimazolam tosylate combined with propofol in upper gastrointestinal endoscopy: A multicenter, randomized clinical trial
Source: PLoS One. 2023 Aug 3;18(8):e0282930. doi: 10.1371/journal.pone.0282930 (PMC10399878; doi:10.1371/journal.pone.0282930)
Supplement: Attachment — Submitted filename: Response to Reviewers .docx [file pone.0282930.s005.docx]

To academic editor:

1. Please ensure that your manuscript meets PLOS ONE's style requirements, including those for file naming.

Reply: Thank you for your reminder. We have revised the manuscript according to the magazine format requirements

2. We note that the status of the clinical trial entry (NCT05429086) is 'not yet recruiting'. Please ensure to update the entry to the current status of the clinical trial.

Reply：We have updated the research information at the Clinical Trials.

3. We note that in the current version of your manuscript Figure 1 is "Fig 1. Duration of blood pressure decrease". Please note that you must upload the completed CONSORT flowchart as figure 1 of your manuscript.

Reply：Information of figure has been modified

4. The information in the registry entry suggests that your trial was registered after patient
   recruitment began. PLOS ONE strongly encourages authors to register all trials before recruiting the first participant in a study.
   As per the journal’s editorial policy, please include in the Methods section of your paper:
   a) your reasons for your delay in registering this study (after enrolment of participants started);
   b) confirmation that all related trials are registered by stating: “The authors confirm that all ongoing and related trials for this drug/intervention are registered”.
   Please also ensure you report the date at which the ethics committee approved the study as well as the complete date range for patient recruitment and follow-up in the Methods section of your manuscript.

Reply: Thank you for your reminder. We have noticed that the recruitment time for patients in the article was before the trial registration. We apologize for the misunderstanding caused by our pen mistake. The actual recruitment time for patients was July 1, 2022, and this information has been modified in the article; We have confirmed that all relevant trials have been registered (Line 98) and reported the time when the ethics committee approved the study, as well as the complete time for recruiting patients and completing follow-up. (Line 101-102)

To Reviewer #1：

1- Abstract: Define all abbreviations at first occurrence

Reply: Already defined. (Line 30-31)

2- Abstract: Provide pairwise comparisons to show which arms differed with respect to hypotension rates.

Reply：Three arms of pairwise comparisons have statistical differences, which have been noted in the article(Line 37-38)

3- Randomization and Blinding: If block randomization was used, indicate the block size.

Reply：Already modified，block of size of 6. (Line 120)

4- Sample size calculation and statistical analysis (p. 8): State the statistical testing method which achieves 80% power.

Reply：A sample size of 229 achieves 80% power to detect an effect size of 0.2051 using a 2 degrees of freedom Chi-Square Test with a significance level (alpha) of 0.05000.(Line 205-207)

5- Sample size calculation and statistical analysis: (p. 9): Specify the nonparametric tests used for quantitative data.

Reply: We have annotated the specific methods of nonparametric testing in the article (Kruskal-Wallis test) (Line 215)

6- Table 1: Explain the type of summary data presented for ASA.

Reply: We have made modifications in the article.(Table 1)

7- State the statistical testing method used for pairwise comparisons when the overall ANOVA was significant. Indicate if chi-square tests used for pairwise comparisons were corrected for multiple comparisons.

Reply: if the difference was significant, the Student-Newman-Keuls q test was further used for pairwise comparison of each group.(Line 216-218). In χ2 test , multiple comparisons of the enumeration data (P group to R group, P group to R+P group, R group to R+P group) were performed, and the α lever was set at 0.017, following Bonferroni adjustment. (Line 219-222)

8- Indicate if adverse events were collected according to a standardized method.

Reply: We have a unified definition for all adverse events and collect them strictly according to the definition, and data is collected uniformly by the evaluation researchers in each center. We have clearly marked it in the manuscript. (Line 179-191)

9- Table 3: Provide the percentages that correspond to the counts.

Reply: Table 3 has added corresponding percentages.

10- Fig 1: Label the units of time on the y-axis.

Reply: Already modified

11- To assist in the review process, add line numbering to the document.

Reply：The manuscript has been added with a line number.

To Reviewer #2:

1- Previous study has reported that Remimazolam Tosylate Combined with Low-Dose Propofol Improves Sedation and Safety in Hysteroscopy (Drug Des Devel Ther. 2022 Nov 29;16:4101-4108.) . What is the difference between the study and your study?

Reply：First of all, our research is different from the purpose of this study. The purpose of this study is to explore the incidence of hypoxemia among the three groups, while our research purpose is to explore the incidence of hypotension among the three groups. Previous studies have shown that hypotension is the most common adverse event when propofol is used for endoscopic sedation, and the degree and duration of Hypotension may be related to organ damage or even mortality in patients（Hypotension during propofol sedation for colonoscopy: a retrospective exploratory analysis and meta-analysis；British Journal of Anaesthesia, 128 (4): 610e622 (2022). Therefore, hypotension should be considered as a serious adverse event in digestive endoscopy, which deserves more clinical attention; Secondly, our research design is more comprehensive. This study is a single center study with a small sample size, while our study is a multicenter study with a larger population and more observed outcome indicators. Our research results include all the results of this study, and we have conducted a deeper exploration of patient recovery and increased satisfaction surveys, which can provide guidance for clinical applications; Finally, our research focuses on the population preparing for gastrointestinal endoscopy, and to our knowledge, there are currently no studies on the combination of remimazolam and propofol in gastrointestinal endoscopy.

2- The adverse events should be listed in the methods of ABSTRACT.

Reply：Adverse events have been added in the summary section. (Line 33-35)

3- MOAA/S score was used to evaluate the anesthesia depth. However, it is difficult to indicate that the anesthesia depth is consistent among groups. Maybe, Bis is better.

Reply: Thank you very much for your suggestion. In response to this issue, the MOAA/S score is currently a commonly used standard for determining the depth of sedation in outpatient settings. It is simple, fast, and can guide clinical medication administration; Secondly, the intravenous Narcotic with the best correlation with BIS is propofol, and benzodiazepines have a worse correlation with BIS than propofol. In our previous research, we explored the correlation between Remimazolam and BIS, but unfortunately, we found that when more patients were given larger doses of Remimazolam, even though the depth of sedation was enough, the BIS value was still high, So we did not consider using BIS for guidance on anesthesia depth in this study.

4- In this study, there are three groups. It means that you have to do χ2 tests twice or three times, so the p values cannot be set at 0.05. It should be 0.025 or 0.017.

Reply：Thank you for your suggestion. The P-value has been corrected. (Line 219-222)
